# Supplementary material for: Association between oral microbiome and seven types of cancers in East Asian population: a two-sample Mendelian randomization analysis
Source: Front Mol Biosci. 2023 Nov 21;10:1327893. doi: 10.3389/fmolb.2023.1327893 (PMC10702768; doi:10.3389/fmolb.2023.1327893)
Supplement: Supplementary file 1 [file DataSheet1.ZIP › Supplementary Materials/MR plots of saliva/saliva═╝/Prostate cancer/pheno.3467_to_prostate cancer_forest.pdf]

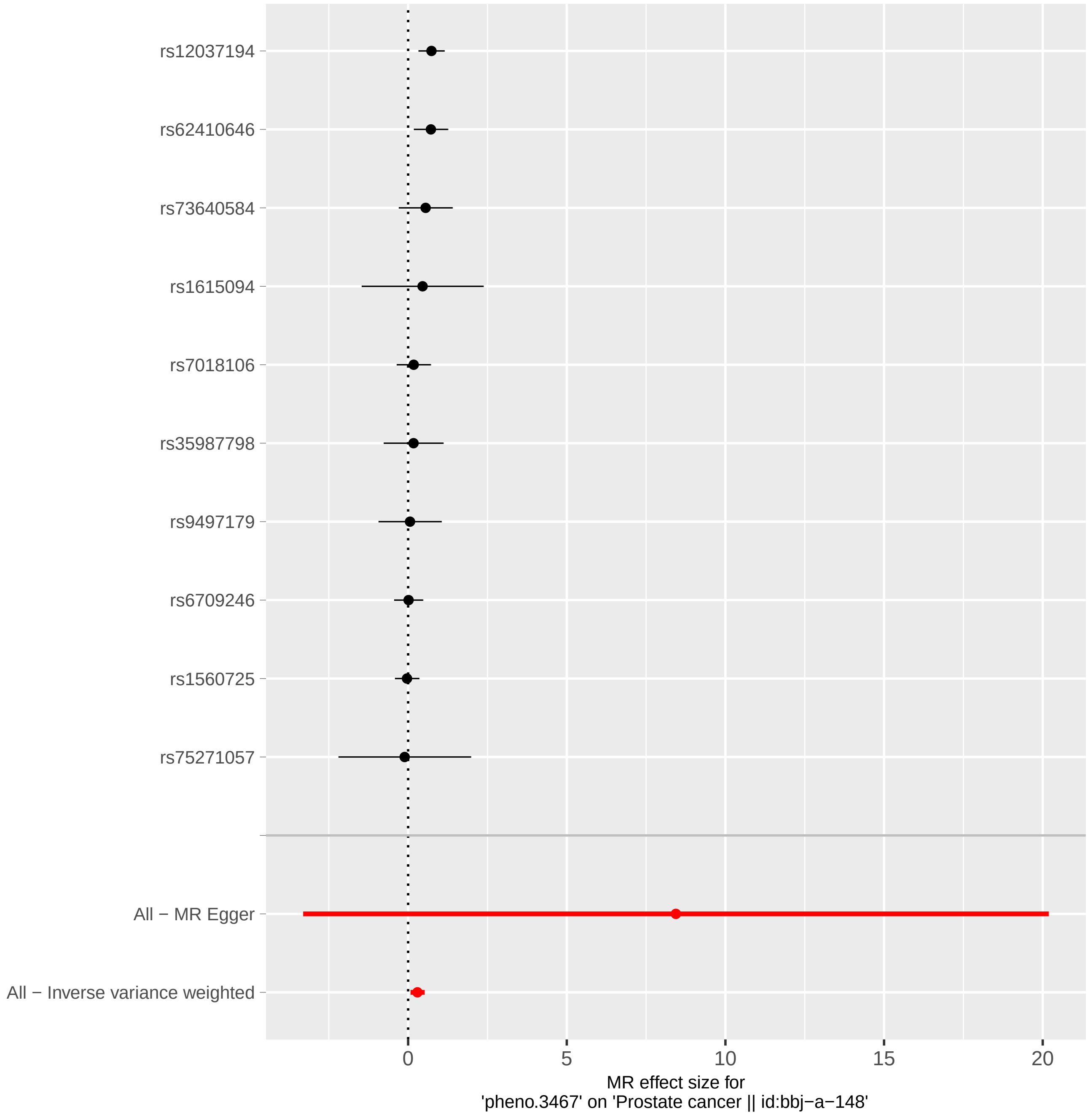

rs12037194
rs62410646
rs73640584
rs1615094
rs7018106
rs35987798
rs9497179
rs6709246
rs1560725
rs75271057
All − MR Egger
All − Inverse variance weighted
0
5
10
15
20
MR effect size for
'pheno.3467' on 'Prostate cancer || id:bbj−a−148'
